# *N*′-[(2-*n*-Butyl-4-chloro-1*H*-imidazol-5-yl)­methyl­idene]adamantane-1-carbo­hydrazide sesquihydrate ethanol hemi­solvate

**DOI:** 10.1107/S1600536810029260

**Published:** 2010-07-31

**Authors:** Abdul-Malek S. Al-Tamimi, Ahmed Bari, Mohamed A. Al-Omar, Ali A. El-Emam, Seik Weng Ng

**Affiliations:** a Department of Pharmaceutical Chemistry, College of Chemistry, King Saud University, Riyadh 11451, Saudi Arabia; b Department of Chemistry, University of Malaya, 50603 Kuala Lumpur, Malaysia

## Abstract

In the asymmetric unit of the title compound, C_19_H_27_ClN_4_O·0.5C_2_H_6_O·1.5H_2_O, there are two mol­ecules of the Schiff base, which has a rigid adamantyl cage at one end of the C(= O)NH–N=CH– chain and an almost planar [torsion angles = 1.3 (1) and  7.9 (2)° imidazolyl ring at the other end, three mol­ecules of water and one mol­ecule of ethanol. In both independent mol­ecules of the Schiff base, this chain displays an extended zigzag configuration. All their amino groups function as hydrogen-bond donors to water mol­ecules; these are linked to other acceptor atoms, generating a layer structure. O—H⋯O and O—H⋯N inter­actions involving the water mol­ecules also occur.

## Related literature

For the cyclization of this class of Schiff bases to pharmaceutically useful chemicals, see: Kadi *et al.* (2007 ▶).
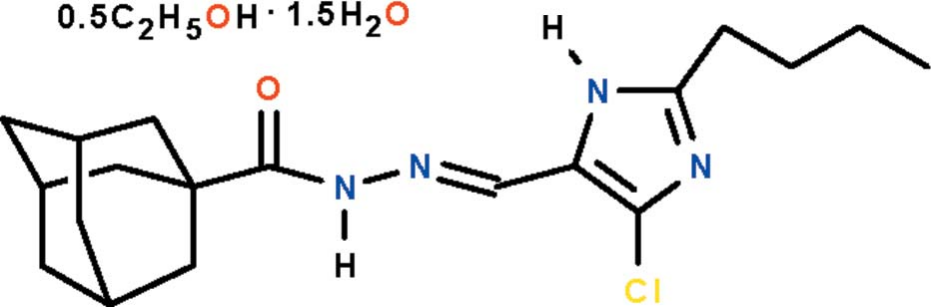


## Experimental

### 

#### Crystal data

C_19_H_27_ClN_4_O·0.5C_2_H_6_O·1.5H_2_O
*M* *_r_* = 412.95
Triclinic, 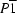

*a* = 7.9867 (6) Å
*b* = 16.8478 (13) Å
*c* = 16.9656 (13) Å
α = 97.341 (1)°
β = 100.376 (1)°
γ = 97.505 (1)°
*V* = 2199.3 (3) Å^3^
*Z* = 4
Mo *K*α radiation
μ = 0.20 mm^−1^
*T* = 100 K
0.40 × 0.10 × 0.10 mm

#### Data collection

Bruker SMART APEX diffractometer
Absorption correction: multi-scan (*SADABS*; Sheldrick, 1996 ▶) *T*_min_ = 0.924, *T*_max_ = 0.980
21291 measured reflections
10050 independent reflections
7547 reflections with *I* > 2σ(*I*)
*R*_int_ = 0.036

#### Refinement

*R*[*F* ^2^ > 2σ(*F* ^2^)] = 0.043
*wR*(*F* ^2^) = 0.113
*S* = 1.02
10050 reflections
552 parameters
11 restraints
H atoms treated by a mixture of independent and constrained refinement
Δρ_max_ = 0.37 e Å^−3^
Δρ_min_ = −0.43 e Å^−3^

### 

Data collection: *APEX2* (Bruker, 2009 ▶); cell refinement: *SAINT* (Bruker, 2009 ▶); data reduction: *SAINT*; program(s) used to solve structure: *SHELXS97* (Sheldrick, 2008 ▶); program(s) used to refine structure: *SHELXL97* (Sheldrick, 2008 ▶); molecular graphics: *X-SEED* (Barbour, 2001 ▶); software used to prepare material for publication: *publCIF* (Westrip, 2010 ▶).

## Supplementary Material

Crystal structure: contains datablocks global, I. DOI: 10.1107/S1600536810029260/bt5304sup1.cif

Structure factors: contains datablocks I. DOI: 10.1107/S1600536810029260/bt5304Isup2.hkl

Additional supplementary materials:  crystallographic information; 3D view; checkCIF report

## Figures and Tables

**Table 1:** Hydrogen-bond geometry (Å, °)

*D*—H⋯*A*	*D*—H	H⋯*A*	*D*⋯*A*	*D*—H⋯*A*
N1—H1⋯O3^i^	0.86 (1)	2.00 (1)	2.841 (2)	166 (2)
N3—H3⋯O1w^ii^	0.86 (1)	1.95 (1)	2.806 (2)	170 (2)
N6—H6⋯O2w	0.88 (1)	1.95 (1)	2.829 (2)	174 (2)
N8—H8⋯O3w^iii^	0.86 (1)	1.94 (1)	2.778 (2)	164 (2)
O3—H3*o*⋯O1w	0.84 (1)	1.84 (1)	2.673 (2)	177 (2)
O1w—H11⋯O1^ii^	0.84 (1)	2.00 (1)	2.821 (2)	166 (2)
O1w—H12⋯N5	0.84 (1)	1.91 (1)	2.751 (2)	175 (3)
O2w—H21⋯O2	0.85 (1)	2.07 (1)	2.905 (2)	172 (2)
O2w—H22⋯O2^iv^	0.84 (1)	1.93 (1)	2.764 (2)	176 (2)
O3w—H31⋯N4	0.84 (1)	1.94 (1)	2.773 (2)	169 (2)
O3w—H32⋯O2w	0.85 (1)	1.92 (1)	2.766 (2)	174 (2)

Symmetry codes: (i) 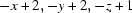; (ii) 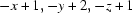; (iii) 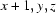; (iv) 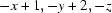.

**Fig. 1.**
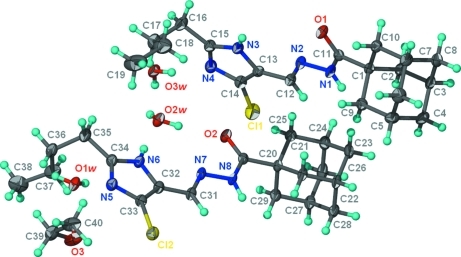
Anisotropic ellipsoid plot (Barbour, 2001) of C19H27ClN4O.0.5C2H5OH.1.5H2O at the 70% probability level; hydrogen atoms are drawn as spheres of arbitrary radius.

**Fig. 2.**
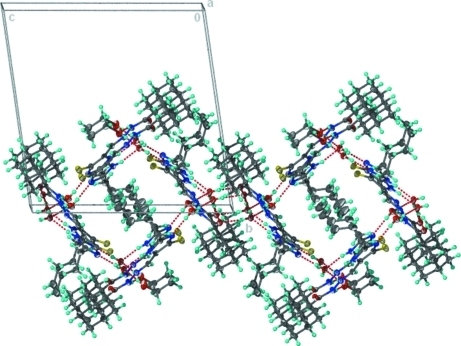
Hydrogen-bonded layer structure.

## supplementary crystallographic information

### Comment

Adamantane-1-carbohydrazide is a commercially available compound that condenses with primary amines to form Schiff bases; some of these Schiff bases can be cyclized to yield pharmaceutically useful compounds (Kadi *et al.*, 2007). However, unlike other aryolhydrazides, there have been no reports of the crystal structures of these Schiff bases. The condensation product with 2-*n*-butyl-5-chloro-1*H*-imidazole-4-carboxaldehyde crystallizes from aqueous ethanol as a sesquihydrate hemiethanol solvate (Scheme 1). There are two independent Schiff base molecules in the asymmetric unit (Fig. 1). The molecule of C_20_H_27_ClN_4_O has a rigid adamantyl cage at one end of the C(═ O)NH–N═CH– chain and a planar imidazolyl ring at the other end; the chain displays an extended zigzag configuration.

The amino groups all function as hydrogen-bond donors to water molecules; these are linked to other acceptor atoms to generate a layer structure (Fig. 2).

### Experimental

Adamantane-1-carbohydrazide (1.94 g, 1 mmol) and 2-*n*-butyl-5-chloro-1*H*-imidazole-4-carboxaldehyde (1.87 g, 1 mmol) were heated in ethanol (50 ml) for 1 h. The solvent was removed and the product recrystallized from aqueous ethanol to afford colorless primatic crystals in 90% yield.

### Refinement

Carbon-bound H-atoms were placed in calculated positions (C—H 0.95 to 0.98 Å) and were included in the refinement in the riding model approximation, with *U*(H) set to 1.2 to 1.5*U*(C).

The amino and water H-atoms were located in a difference Fourier map, and were refined isotropically with distance restraints of N–H 0.86±0.01 Å and O–H 0.84±0.01 Å.

### Crystal data

**Table:**

C_19_H_27_ClN_4_O·0.5C_2_H_6_O·1.5H_2_O	*Z* = 4
*M**_r_* = 412.95	*F*(000) = 888
Triclinic, *P*1	*D*_x_ = 1.247 Mg m^−^^3^
Hall symbol: -P 1	Mo *K*α radiation, λ = 0.71073 Å
*a* = 7.9867 (6) Å	Cell parameters from 4692 reflections
*b* = 16.8478 (13) Å	θ = 2.5–28.2°
*c* = 16.9656 (13) Å	µ = 0.20 mm^−^^1^
α = 97.341 (1)°	*T* = 100 K
β = 100.376 (1)°	Prism, colorless
γ = 97.505 (1)°	0.40 × 0.10 × 0.10 mm
*V* = 2199.3 (3) Å^3^	

### Data collection

**Table:**

Bruker SMART APEX diffractometer	10050 independent reflections
Radiation source: fine-focus sealed tube	7547 reflections with *I* > 2σ(*I*)
graphite	*R*_int_ = 0.036
ω scans	θ_max_ = 27.5°, θ_min_ = 1.6°
Absorption correction: multi-scan (*SADABS*; Sheldrick, 1996)	*h* = −10→10
*T*_min_ = 0.924, *T*_max_ = 0.980	*k* = −22→21
21291 measured reflections	*l* = −21→21

### Refinement

**Table:**

Refinement on *F*^2^	Primary atom site location: structure-invariant direct methods
Least-squares matrix: full	Secondary atom site location: difference Fourier map
*R*[*F*^2^ > 2σ(*F*^2^)] = 0.043	Hydrogen site location: inferred from neighbouring sites
*wR*(*F*^2^) = 0.113	H atoms treated by a mixture of independent and constrained refinement
*S* = 1.02	*w* = 1/[σ^2^(*F*_o_^2^) + (0.0579*P*)^2^] where *P* = (*F*_o_^2^ + 2*F*_c_^2^)/3
10050 reflections	(Δ/σ)_max_ = 0.001
552 parameters	Δρ_max_ = 0.37 e Å^−^^3^
11 restraints	Δρ_min_ = −0.43 e Å^−^^3^

### Fractional atomic coordinates and isotropic or equivalent isotropic displacement parameters (Å^2^)

**Table:**

	*x*	*y*	*z*	*U*_iso_*/*U*_eq_	
Cl1	0.76837 (6)	1.16482 (3)	0.27186 (3)	0.02855 (12)	
Cl2	0.96970 (5)	0.80656 (2)	0.35728 (3)	0.02268 (10)	
O1	0.75877 (14)	1.40939 (7)	0.65640 (7)	0.0201 (3)	
O2	0.73265 (14)	1.07788 (7)	0.06758 (7)	0.0188 (2)	
O3	0.78300 (16)	0.63333 (8)	0.50491 (8)	0.0284 (3)	
O1W	0.54116 (15)	0.70102 (7)	0.41892 (7)	0.0196 (3)	
O2W	0.46191 (15)	0.95403 (7)	0.08834 (7)	0.0201 (3)	
O3W	0.27823 (15)	1.00424 (7)	0.20395 (7)	0.0216 (3)	
N1	0.92004 (18)	1.38006 (8)	0.56315 (8)	0.0180 (3)	
N2	0.78186 (17)	1.32710 (8)	0.51751 (8)	0.0178 (3)	
N3	0.50609 (18)	1.21097 (8)	0.43693 (8)	0.0173 (3)	
N4	0.46659 (18)	1.13115 (8)	0.31913 (8)	0.0204 (3)	
N5	0.62851 (18)	0.77835 (8)	0.29533 (8)	0.0195 (3)	
N6	0.60000 (18)	0.86304 (8)	0.20631 (8)	0.0176 (3)	
N7	0.83450 (17)	0.98033 (8)	0.16998 (8)	0.0158 (3)	
N8	0.95038 (17)	1.04002 (8)	0.15190 (8)	0.0151 (3)	
C1	1.0617 (2)	1.46542 (9)	0.69156 (9)	0.0157 (3)	
C2	1.1519 (2)	1.52998 (9)	0.64919 (10)	0.0182 (3)	
H2A	1.1853	1.5032	0.6002	0.022*	
H2B	1.0716	1.5675	0.6321	0.022*	
C3	1.3131 (2)	1.57772 (10)	0.70820 (10)	0.0214 (4)	
H3A	1.3706	1.6198	0.6811	0.026*	
C4	1.4376 (2)	1.51865 (11)	0.73189 (11)	0.0238 (4)	
H4A	1.4708	1.4921	0.6828	0.029*	
H4B	1.5435	1.5487	0.7688	0.029*	
C5	1.3512 (2)	1.45452 (10)	0.77393 (10)	0.0218 (4)	
H5	1.4330	1.4164	0.7895	0.026*	
C6	1.3012 (2)	1.49608 (11)	0.85039 (10)	0.0250 (4)	
H6A	1.2465	1.4548	0.8783	0.030*	
H6B	1.4057	1.5264	0.8881	0.030*	
C7	1.1755 (2)	1.55423 (10)	0.82642 (10)	0.0218 (4)	
H7	1.1428	1.5813	0.8762	0.026*	
C8	1.2614 (2)	1.61865 (10)	0.78384 (11)	0.0249 (4)	
H8A	1.1803	1.6563	0.7684	0.030*	
H8B	1.3648	1.6504	0.8213	0.030*	
C9	1.1886 (2)	1.40708 (10)	0.71633 (10)	0.0184 (3)	
H9A	1.1329	1.3654	0.7435	0.022*	
H9B	1.2203	1.3793	0.6674	0.022*	
C10	1.0134 (2)	1.50723 (10)	0.76836 (10)	0.0183 (3)	
H10A	0.9319	1.5449	0.7534	0.022*	
H10B	0.9559	1.4661	0.7956	0.022*	
C11	0.8996 (2)	1.41698 (9)	0.63612 (9)	0.0153 (3)	
C12	0.8028 (2)	1.28451 (9)	0.45310 (9)	0.0173 (3)	
H12A	0.9093	1.2909	0.4354	0.021*	
C13	0.6573 (2)	1.22641 (9)	0.40851 (10)	0.0165 (3)	
C14	0.6272 (2)	1.17570 (10)	0.33610 (10)	0.0185 (3)	
C15	0.3969 (2)	1.15408 (10)	0.38198 (10)	0.0198 (4)	
C16	0.2199 (2)	1.11990 (11)	0.39062 (11)	0.0251 (4)	
H16A	0.1425	1.1094	0.3366	0.030*	
H16B	0.1759	1.1605	0.4256	0.030*	
C17	0.2146 (2)	1.04091 (11)	0.42715 (12)	0.0308 (4)	
H17A	0.0940	1.0127	0.4158	0.037*	
H17B	0.2833	1.0051	0.4003	0.037*	
C18	0.2832 (3)	1.05454 (13)	0.51753 (13)	0.0377 (5)	
H18A	0.2149	1.0907	0.5443	0.045*	
H18B	0.4039	1.0825	0.5287	0.045*	
C19	0.2776 (3)	0.97727 (16)	0.55442 (17)	0.0588 (8)	
H19A	0.3189	0.9906	0.6133	0.088*	
H19B	0.3515	0.9426	0.5311	0.088*	
H19C	0.1589	0.9486	0.5428	0.088*	
C20	1.0200 (2)	1.15241 (9)	0.07997 (9)	0.0143 (3)	
C21	1.1515 (2)	1.19510 (10)	0.15687 (9)	0.0173 (3)	
H21A	1.0903	1.2215	0.1962	0.021*	
H21B	1.2121	1.1546	0.1827	0.021*	
C22	1.2828 (2)	1.25884 (10)	0.13452 (10)	0.0191 (4)	
H22A	1.3672	1.2860	0.1846	0.023*	
C23	1.1891 (2)	1.32202 (10)	0.09564 (11)	0.0239 (4)	
H23A	1.1275	1.3491	0.1344	0.029*	
H23B	1.2736	1.3637	0.0818	0.029*	
C24	1.0604 (2)	1.28048 (10)	0.01893 (11)	0.0233 (4)	
H24	0.9992	1.3218	−0.0064	0.028*	
C25	0.9281 (2)	1.21643 (10)	0.04083 (11)	0.0201 (4)	
H25A	0.8645	1.2429	0.0790	0.024*	
H25B	0.8439	1.1900	−0.0088	0.024*	
C26	1.1560 (2)	1.23945 (11)	−0.04119 (11)	0.0259 (4)	
H26A	1.0730	1.2130	−0.0911	0.031*	
H26B	1.2402	1.2805	−0.0562	0.031*	
C27	1.2497 (2)	1.17624 (10)	−0.00257 (10)	0.0206 (4)	
H27	1.3125	1.1496	−0.0419	0.025*	
C28	1.3788 (2)	1.21725 (10)	0.07450 (10)	0.0216 (4)	
H28A	1.4657	1.2579	0.0607	0.026*	
H28B	1.4395	1.1763	0.0996	0.026*	
C29	1.1182 (2)	1.11208 (9)	0.01915 (10)	0.0181 (3)	
H29A	1.1784	1.0706	0.0435	0.022*	
H29B	1.0355	1.0849	−0.0306	0.022*	
C30	0.8874 (2)	1.08749 (9)	0.09866 (9)	0.0147 (3)	
C31	0.8957 (2)	0.93466 (9)	0.21920 (9)	0.0170 (3)	
H31A	1.0157	0.9405	0.2410	0.020*	
C32	0.7734 (2)	0.87396 (9)	0.23988 (9)	0.0168 (3)	
C33	0.7857 (2)	0.82064 (10)	0.29406 (10)	0.0182 (3)	
C34	0.5179 (2)	0.80624 (10)	0.24081 (10)	0.0188 (3)	
C35	0.3269 (2)	0.77994 (10)	0.21929 (11)	0.0235 (4)	
H35A	0.2761	0.8135	0.1804	0.028*	
H35B	0.2786	0.7898	0.2688	0.028*	
C36	0.2740 (2)	0.69046 (10)	0.18198 (10)	0.0223 (4)	
H36A	0.3137	0.6569	0.2233	0.027*	
H36B	0.1466	0.6782	0.1680	0.027*	
C37	0.3442 (2)	0.66585 (11)	0.10660 (11)	0.0273 (4)	
H37A	0.4717	0.6740	0.1209	0.033*	
H37B	0.3106	0.7011	0.0660	0.033*	
C38	0.2775 (3)	0.57763 (13)	0.06950 (12)	0.0390 (5)	
H38A	0.3292	0.5636	0.0224	0.058*	
H38B	0.1518	0.5700	0.0523	0.058*	
H38C	0.3085	0.5426	0.1099	0.058*	
C39	0.7041 (2)	0.59637 (11)	0.56315 (11)	0.0292 (4)	
H39A	0.7416	0.5431	0.5676	0.035*	
H39B	0.5773	0.5871	0.5446	0.035*	
C40	0.7512 (3)	0.64869 (12)	0.64503 (11)	0.0317 (4)	
H40A	0.7063	0.6193	0.6850	0.048*	
H40B	0.7011	0.6986	0.6423	0.048*	
H40C	0.8769	0.6623	0.6611	0.048*	
H3o	0.709 (2)	0.6547 (12)	0.4769 (11)	0.040 (6)*	
H11	0.4462 (17)	0.6699 (11)	0.4039 (12)	0.039 (6)*	
H12	0.561 (3)	0.7241 (15)	0.3798 (11)	0.068 (9)*	
H21	0.547 (2)	0.9885 (11)	0.0863 (14)	0.047 (7)*	
H22	0.402 (2)	0.9418 (11)	0.0416 (7)	0.028 (5)*	
H31	0.348 (2)	1.0401 (11)	0.2379 (11)	0.050 (7)*	
H32	0.328 (3)	0.9889 (13)	0.1658 (10)	0.047 (7)*	
H1	1.0165 (16)	1.3843 (11)	0.5468 (11)	0.022 (5)*	
H3	0.491 (2)	1.2324 (11)	0.4835 (7)	0.030 (5)*	
H6	0.553 (2)	0.8878 (11)	0.1671 (9)	0.032 (6)*	
H8	1.0584 (13)	1.0385 (11)	0.1682 (11)	0.029 (5)*	

### Atomic displacement parameters (Å^2^)

**Table:**

	*U*^11^	*U*^22^	*U*^33^	*U*^12^	*U*^13^	*U*^23^
Cl1	0.0305 (3)	0.0324 (2)	0.0219 (2)	0.00006 (19)	0.01191 (19)	−0.00352 (18)
Cl2	0.0199 (2)	0.0231 (2)	0.0234 (2)	0.00125 (16)	−0.00039 (17)	0.00668 (16)
O1	0.0146 (6)	0.0253 (6)	0.0187 (6)	−0.0019 (5)	0.0044 (5)	0.0003 (5)
O2	0.0139 (6)	0.0229 (6)	0.0171 (6)	−0.0021 (5)	−0.0006 (5)	0.0038 (5)
O3	0.0208 (7)	0.0456 (8)	0.0228 (7)	0.0111 (6)	0.0065 (6)	0.0107 (6)
O1W	0.0161 (6)	0.0242 (6)	0.0177 (6)	−0.0031 (5)	0.0048 (5)	0.0044 (5)
O2W	0.0151 (6)	0.0263 (7)	0.0163 (6)	−0.0022 (5)	−0.0003 (5)	0.0045 (5)
O3W	0.0147 (6)	0.0240 (6)	0.0226 (7)	−0.0009 (5)	0.0018 (5)	−0.0030 (5)
N1	0.0125 (7)	0.0218 (7)	0.0166 (7)	−0.0038 (6)	0.0030 (6)	−0.0012 (6)
N2	0.0152 (7)	0.0191 (7)	0.0162 (7)	−0.0024 (5)	0.0006 (6)	0.0007 (5)
N3	0.0159 (7)	0.0194 (7)	0.0155 (7)	0.0012 (6)	0.0029 (6)	0.0009 (6)
N4	0.0193 (8)	0.0203 (7)	0.0191 (7)	0.0003 (6)	0.0003 (6)	0.0012 (6)
N5	0.0180 (7)	0.0199 (7)	0.0211 (7)	0.0002 (6)	0.0058 (6)	0.0052 (6)
N6	0.0143 (7)	0.0188 (7)	0.0197 (7)	−0.0002 (6)	0.0036 (6)	0.0053 (6)
N7	0.0151 (7)	0.0164 (7)	0.0146 (7)	−0.0035 (5)	0.0047 (5)	0.0010 (5)
N8	0.0111 (7)	0.0167 (7)	0.0159 (7)	−0.0026 (5)	0.0021 (5)	0.0029 (5)
C1	0.0137 (8)	0.0160 (8)	0.0161 (8)	0.0000 (6)	0.0015 (6)	0.0015 (6)
C2	0.0155 (8)	0.0178 (8)	0.0210 (8)	0.0000 (6)	0.0035 (7)	0.0047 (7)
C3	0.0157 (8)	0.0199 (8)	0.0259 (9)	−0.0040 (7)	0.0034 (7)	0.0018 (7)
C4	0.0138 (8)	0.0291 (9)	0.0248 (9)	0.0008 (7)	0.0008 (7)	−0.0030 (7)
C5	0.0185 (9)	0.0260 (9)	0.0191 (9)	0.0073 (7)	−0.0019 (7)	0.0011 (7)
C6	0.0248 (10)	0.0287 (9)	0.0175 (9)	0.0023 (8)	−0.0011 (7)	−0.0017 (7)
C7	0.0182 (9)	0.0241 (9)	0.0202 (9)	0.0012 (7)	0.0033 (7)	−0.0049 (7)
C8	0.0185 (9)	0.0193 (8)	0.0313 (10)	−0.0011 (7)	−0.0010 (8)	−0.0042 (7)
C9	0.0188 (9)	0.0167 (8)	0.0182 (8)	0.0019 (6)	0.0012 (7)	0.0010 (6)
C10	0.0169 (8)	0.0191 (8)	0.0175 (8)	0.0008 (7)	0.0037 (7)	−0.0011 (6)
C11	0.0165 (8)	0.0144 (7)	0.0156 (8)	0.0017 (6)	0.0038 (6)	0.0042 (6)
C12	0.0154 (8)	0.0193 (8)	0.0169 (8)	0.0000 (6)	0.0045 (7)	0.0034 (6)
C13	0.0147 (8)	0.0184 (8)	0.0171 (8)	0.0027 (6)	0.0038 (6)	0.0040 (6)
C14	0.0198 (9)	0.0208 (8)	0.0150 (8)	0.0022 (7)	0.0039 (7)	0.0032 (6)
C15	0.0170 (9)	0.0197 (8)	0.0207 (9)	0.0015 (7)	−0.0001 (7)	0.0030 (7)
C16	0.0147 (9)	0.0299 (10)	0.0275 (10)	−0.0006 (7)	0.0004 (7)	0.0021 (8)
C17	0.0235 (10)	0.0261 (10)	0.0420 (12)	−0.0028 (8)	0.0130 (9)	0.0003 (8)
C18	0.0370 (12)	0.0422 (12)	0.0415 (12)	0.0121 (10)	0.0177 (10)	0.0152 (10)
C19	0.0459 (15)	0.0676 (17)	0.088 (2)	0.0273 (13)	0.0378 (14)	0.0514 (16)
C20	0.0138 (8)	0.0145 (7)	0.0128 (7)	−0.0010 (6)	0.0009 (6)	0.0010 (6)
C21	0.0155 (8)	0.0184 (8)	0.0151 (8)	−0.0037 (6)	0.0021 (6)	−0.0004 (6)
C22	0.0159 (8)	0.0181 (8)	0.0192 (8)	−0.0048 (6)	0.0016 (7)	−0.0015 (6)
C23	0.0231 (9)	0.0150 (8)	0.0320 (10)	−0.0036 (7)	0.0092 (8)	−0.0007 (7)
C24	0.0201 (9)	0.0187 (8)	0.0302 (10)	−0.0001 (7)	0.0007 (8)	0.0098 (7)
C25	0.0159 (8)	0.0188 (8)	0.0255 (9)	0.0022 (7)	0.0027 (7)	0.0057 (7)
C26	0.0273 (10)	0.0272 (9)	0.0201 (9)	−0.0100 (8)	0.0039 (8)	0.0077 (7)
C27	0.0206 (9)	0.0203 (8)	0.0203 (9)	−0.0026 (7)	0.0104 (7)	−0.0014 (7)
C28	0.0162 (8)	0.0215 (8)	0.0267 (9)	−0.0025 (7)	0.0074 (7)	0.0031 (7)
C29	0.0191 (9)	0.0168 (8)	0.0168 (8)	0.0000 (6)	0.0048 (7)	−0.0020 (6)
C30	0.0155 (8)	0.0148 (7)	0.0120 (7)	0.0001 (6)	0.0025 (6)	−0.0013 (6)
C31	0.0157 (8)	0.0186 (8)	0.0150 (8)	−0.0015 (6)	0.0028 (6)	0.0008 (6)
C32	0.0162 (8)	0.0182 (8)	0.0142 (8)	−0.0006 (6)	0.0017 (6)	0.0007 (6)
C33	0.0177 (8)	0.0190 (8)	0.0175 (8)	0.0016 (7)	0.0032 (7)	0.0026 (6)
C34	0.0188 (9)	0.0177 (8)	0.0207 (8)	0.0005 (7)	0.0074 (7)	0.0035 (7)
C35	0.0183 (9)	0.0240 (9)	0.0292 (10)	−0.0001 (7)	0.0077 (7)	0.0080 (7)
C36	0.0183 (9)	0.0238 (9)	0.0248 (9)	−0.0013 (7)	0.0040 (7)	0.0085 (7)
C37	0.0262 (10)	0.0362 (10)	0.0208 (9)	0.0086 (8)	0.0015 (8)	0.0095 (8)
C38	0.0404 (13)	0.0435 (12)	0.0278 (11)	0.0141 (10)	−0.0064 (9)	−0.0027 (9)
C39	0.0210 (10)	0.0311 (10)	0.0358 (11)	0.0006 (8)	0.0022 (8)	0.0149 (8)
C40	0.0294 (11)	0.0440 (12)	0.0294 (10)	0.0146 (9)	0.0119 (8)	0.0166 (9)

### Geometric parameters (Å, °)

**Table:**

Cl1—C14	1.7142 (17)	C16—C17	1.537 (3)
Cl2—C33	1.7186 (17)	C16—H16A	0.9900
O1—C11	1.2300 (19)	C16—H16B	0.9900
O2—C30	1.2344 (19)	C17—C18	1.510 (3)
O3—C39	1.428 (2)	C17—H17A	0.9900
O3—H3o	0.84 (1)	C17—H17B	0.9900
O1W—H11	0.84 (1)	C18—C19	1.514 (3)
O1W—H12	0.84 (1)	C18—H18A	0.9900
O2W—H21	0.85 (1)	C18—H18B	0.9900
O2W—H22	0.84 (1)	C19—H19A	0.9800
O3W—H31	0.84 (1)	C19—H19B	0.9800
O3W—H32	0.85 (1)	C19—H19C	0.9800
N1—C11	1.360 (2)	C20—C30	1.525 (2)
N1—N2	1.3682 (18)	C20—C25	1.535 (2)
N1—H1	0.86 (1)	C20—C21	1.547 (2)
N2—C12	1.278 (2)	C20—C29	1.547 (2)
N3—C15	1.345 (2)	C21—C22	1.533 (2)
N3—C13	1.385 (2)	C21—H21A	0.9900
N3—H3	0.86 (1)	C21—H21B	0.9900
N4—C15	1.327 (2)	C22—C23	1.529 (2)
N4—C14	1.361 (2)	C22—C28	1.535 (2)
N5—C34	1.335 (2)	C22—H22A	1.0000
N5—C33	1.366 (2)	C23—C24	1.529 (2)
N6—C34	1.344 (2)	C23—H23A	0.9900
N6—C32	1.378 (2)	C23—H23B	0.9900
N6—H6	0.88 (1)	C24—C26	1.529 (3)
N7—C31	1.282 (2)	C24—C25	1.538 (2)
N7—N8	1.3784 (18)	C24—H24	1.0000
N8—C30	1.354 (2)	C25—H25A	0.9900
N8—H8	0.86 (1)	C25—H25B	0.9900
C1—C11	1.522 (2)	C26—C27	1.527 (2)
C1—C10	1.536 (2)	C26—H26A	0.9900
C1—C9	1.544 (2)	C26—H26B	0.9900
C1—C2	1.549 (2)	C27—C28	1.532 (2)
C2—C3	1.538 (2)	C27—C29	1.533 (2)
C2—H2A	0.9900	C27—H27	1.0000
C2—H2B	0.9900	C28—H28A	0.9900
C3—C8	1.527 (2)	C28—H28B	0.9900
C3—C4	1.536 (2)	C29—H29A	0.9900
C3—H3A	1.0000	C29—H29B	0.9900
C4—C5	1.527 (2)	C31—C32	1.441 (2)
C4—H4A	0.9900	C31—H31A	0.9500
C4—H4B	0.9900	C32—C33	1.365 (2)
C5—C9	1.534 (2)	C34—C35	1.496 (2)
C5—C6	1.536 (2)	C35—C36	1.531 (2)
C5—H5	1.0000	C35—H35A	0.9900
C6—C7	1.531 (2)	C35—H35B	0.9900
C6—H6A	0.9900	C36—C37	1.519 (2)
C6—H6B	0.9900	C36—H36A	0.9900
C7—C10	1.532 (2)	C36—H36B	0.9900
C7—C8	1.535 (2)	C37—C38	1.524 (3)
C7—H7	1.0000	C37—H37A	0.9900
C8—H8A	0.9900	C37—H37B	0.9900
C8—H8B	0.9900	C38—H38A	0.9800
C9—H9A	0.9900	C38—H38B	0.9800
C9—H9B	0.9900	C38—H38C	0.9800
C10—H10A	0.9900	C39—C40	1.503 (3)
C10—H10B	0.9900	C39—H39A	0.9900
C12—C13	1.441 (2)	C39—H39B	0.9900
C12—H12A	0.9500	C40—H40A	0.9800
C13—C14	1.367 (2)	C40—H40B	0.9800
C15—C16	1.494 (2)	C40—H40C	0.9800
			
C39—O3—H3o	108.2 (15)	H19A—C19—H19B	109.5
H11—O1W—H12	108 (2)	C18—C19—H19C	109.5
H21—O2W—H22	107 (2)	H19A—C19—H19C	109.5
H31—O3W—H32	109 (2)	H19B—C19—H19C	109.5
C11—N1—N2	116.73 (13)	C30—C20—C25	109.75 (13)
C11—N1—H1	124.0 (13)	C30—C20—C21	112.26 (12)
N2—N1—H1	119.0 (12)	C25—C20—C21	108.69 (13)
C12—N2—N1	118.29 (14)	C30—C20—C29	108.65 (12)
C15—N3—C13	107.94 (14)	C25—C20—C29	108.58 (13)
C15—N3—H3	127.7 (13)	C21—C20—C29	108.83 (13)
C13—N3—H3	124.2 (13)	C22—C21—C20	110.09 (13)
C15—N4—C14	104.80 (13)	C22—C21—H21A	109.6
C34—N5—C33	104.56 (13)	C20—C21—H21A	109.6
C34—N6—C32	108.41 (14)	C22—C21—H21B	109.6
C34—N6—H6	126.6 (13)	C20—C21—H21B	109.6
C32—N6—H6	125.0 (13)	H21A—C21—H21B	108.2
C31—N7—N8	116.86 (14)	C23—C22—C28	109.68 (14)
C30—N8—N7	117.28 (13)	C23—C22—C21	109.51 (14)
C30—N8—H8	124.6 (13)	C28—C22—C21	109.28 (13)
N7—N8—H8	117.2 (13)	C23—C22—H22A	109.5
C11—C1—C10	109.09 (13)	C28—C22—H22A	109.5
C11—C1—C9	109.09 (12)	C21—C22—H22A	109.5
C10—C1—C9	108.73 (13)	C24—C23—C22	109.42 (13)
C11—C1—C2	111.73 (13)	C24—C23—H23A	109.8
C10—C1—C2	109.51 (13)	C22—C23—H23A	109.8
C9—C1—C2	108.64 (13)	C24—C23—H23B	109.8
C3—C2—C1	109.50 (13)	C22—C23—H23B	109.8
C3—C2—H2A	109.8	H23A—C23—H23B	108.2
C1—C2—H2A	109.8	C23—C24—C26	109.71 (14)
C3—C2—H2B	109.8	C23—C24—C25	109.66 (14)
C1—C2—H2B	109.8	C26—C24—C25	109.38 (14)
H2A—C2—H2B	108.2	C23—C24—H24	109.4
C8—C3—C4	109.85 (14)	C26—C24—H24	109.4
C8—C3—C2	109.70 (14)	C25—C24—H24	109.4
C4—C3—C2	108.74 (13)	C20—C25—C24	110.06 (13)
C8—C3—H3A	109.5	C20—C25—H25A	109.6
C4—C3—H3A	109.5	C24—C25—H25A	109.6
C2—C3—H3A	109.5	C20—C25—H25B	109.6
C5—C4—C3	109.80 (14)	C24—C25—H25B	109.6
C5—C4—H4A	109.7	H25A—C25—H25B	108.2
C3—C4—H4A	109.7	C27—C26—C24	109.48 (14)
C5—C4—H4B	109.7	C27—C26—H26A	109.8
C3—C4—H4B	109.7	C24—C26—H26A	109.8
H4A—C4—H4B	108.2	C27—C26—H26B	109.8
C4—C5—C9	109.74 (13)	C24—C26—H26B	109.8
C4—C5—C6	109.39 (14)	H26A—C26—H26B	108.2
C9—C5—C6	109.32 (14)	C26—C27—C28	109.75 (14)
C4—C5—H5	109.5	C26—C27—C29	109.49 (14)
C9—C5—H5	109.5	C28—C27—C29	109.40 (13)
C6—C5—H5	109.5	C26—C27—H27	109.4
C7—C6—C5	109.40 (14)	C28—C27—H27	109.4
C7—C6—H6A	109.8	C29—C27—H27	109.4
C5—C6—H6A	109.8	C27—C28—C22	109.47 (13)
C7—C6—H6B	109.8	C27—C28—H28A	109.8
C5—C6—H6B	109.8	C22—C28—H28A	109.8
H6A—C6—H6B	108.2	C27—C28—H28B	109.8
C6—C7—C10	109.82 (14)	C22—C28—H28B	109.8
C6—C7—C8	109.62 (14)	H28A—C28—H28B	108.2
C10—C7—C8	108.98 (14)	C27—C29—C20	109.95 (12)
C6—C7—H7	109.5	C27—C29—H29A	109.7
C10—C7—H7	109.5	C20—C29—H29A	109.7
C8—C7—H7	109.5	C27—C29—H29B	109.7
C3—C8—C7	109.75 (13)	C20—C29—H29B	109.7
C3—C8—H8A	109.7	H29A—C29—H29B	108.2
C7—C8—H8A	109.7	O2—C30—N8	121.49 (14)
C3—C8—H8B	109.7	O2—C30—C20	122.90 (14)
C7—C8—H8B	109.7	N8—C30—C20	115.61 (13)
H8A—C8—H8B	108.2	N7—C31—C32	116.67 (15)
C5—C9—C1	109.88 (13)	N7—C31—H31A	121.7
C5—C9—H9A	109.7	C32—C31—H31A	121.7
C1—C9—H9A	109.7	C33—C32—N6	104.13 (14)
C5—C9—H9B	109.7	C33—C32—C31	133.72 (16)
C1—C9—H9B	109.7	N6—C32—C31	122.04 (15)
H9A—C9—H9B	108.2	C32—C33—N5	111.78 (15)
C7—C10—C1	110.06 (13)	C32—C33—Cl2	126.87 (13)
C7—C10—H10A	109.6	N5—C33—Cl2	121.32 (12)
C1—C10—H10A	109.6	N5—C34—N6	111.12 (15)
C7—C10—H10B	109.6	N5—C34—C35	126.11 (15)
C1—C10—H10B	109.6	N6—C34—C35	122.77 (15)
H10A—C10—H10B	108.2	C34—C35—C36	113.43 (14)
O1—C11—N1	121.44 (15)	C34—C35—H35A	108.9
O1—C11—C1	122.50 (14)	C36—C35—H35A	108.9
N1—C11—C1	116.03 (14)	C34—C35—H35B	108.9
N2—C12—C13	116.98 (15)	C36—C35—H35B	108.9
N2—C12—H12A	121.5	H35A—C35—H35B	107.7
C13—C12—H12A	121.5	C37—C36—C35	114.70 (14)
C14—C13—N3	103.96 (14)	C37—C36—H36A	108.6
C14—C13—C12	133.60 (15)	C35—C36—H36A	108.6
N3—C13—C12	122.43 (14)	C37—C36—H36B	108.6
N4—C14—C13	111.82 (14)	C35—C36—H36B	108.6
N4—C14—Cl1	121.52 (12)	H36A—C36—H36B	107.6
C13—C14—Cl1	126.65 (13)	C36—C37—C38	111.99 (16)
N4—C15—N3	111.47 (14)	C36—C37—H37A	109.2
N4—C15—C16	123.95 (15)	C38—C37—H37A	109.2
N3—C15—C16	124.58 (15)	C36—C37—H37B	109.2
C15—C16—C17	113.03 (15)	C38—C37—H37B	109.2
C15—C16—H16A	109.0	H37A—C37—H37B	107.9
C17—C16—H16A	109.0	C37—C38—H38A	109.5
C15—C16—H16B	109.0	C37—C38—H38B	109.5
C17—C16—H16B	109.0	H38A—C38—H38B	109.5
H16A—C16—H16B	107.8	C37—C38—H38C	109.5
C18—C17—C16	113.12 (16)	H38A—C38—H38C	109.5
C18—C17—H17A	109.0	H38B—C38—H38C	109.5
C16—C17—H17A	109.0	O3—C39—C40	111.29 (15)
C18—C17—H17B	109.0	O3—C39—H39A	109.4
C16—C17—H17B	109.0	C40—C39—H39A	109.4
H17A—C17—H17B	107.8	O3—C39—H39B	109.4
C17—C18—C19	113.7 (2)	C40—C39—H39B	109.4
C17—C18—H18A	108.8	H39A—C39—H39B	108.0
C19—C18—H18A	108.8	C39—C40—H40A	109.5
C17—C18—H18B	108.8	C39—C40—H40B	109.5
C19—C18—H18B	108.8	H40A—C40—H40B	109.5
H18A—C18—H18B	107.7	C39—C40—H40C	109.5
C18—C19—H19A	109.5	H40A—C40—H40C	109.5
C18—C19—H19B	109.5	H40B—C40—H40C	109.5
			
C11—N1—N2—C12	−172.14 (15)	C16—C17—C18—C19	179.65 (17)
C31—N7—N8—C30	178.67 (14)	C30—C20—C21—C22	179.26 (13)
C11—C1—C2—C3	179.27 (13)	C25—C20—C21—C22	−59.15 (17)
C10—C1—C2—C3	58.29 (17)	C29—C20—C21—C22	58.94 (17)
C9—C1—C2—C3	−60.33 (16)	C20—C21—C22—C23	60.07 (17)
C1—C2—C3—C8	−59.13 (17)	C20—C21—C22—C28	−60.09 (17)
C1—C2—C3—C4	61.03 (17)	C28—C22—C23—C24	59.73 (17)
C8—C3—C4—C5	59.27 (17)	C21—C22—C23—C24	−60.19 (17)
C2—C3—C4—C5	−60.79 (17)	C22—C23—C24—C26	−60.06 (17)
C3—C4—C5—C9	60.18 (17)	C22—C23—C24—C25	60.10 (18)
C3—C4—C5—C6	−59.74 (17)	C30—C20—C25—C24	−177.94 (13)
C4—C5—C6—C7	60.22 (18)	C21—C20—C25—C24	58.94 (17)
C9—C5—C6—C7	−59.97 (18)	C29—C20—C25—C24	−59.30 (17)
C5—C6—C7—C10	59.72 (18)	C23—C24—C25—C20	−60.05 (18)
C5—C6—C7—C8	−59.99 (18)	C26—C24—C25—C20	60.30 (18)
C4—C3—C8—C7	−58.87 (18)	C23—C24—C26—C27	60.04 (17)
C2—C3—C8—C7	60.61 (18)	C25—C24—C26—C27	−60.28 (18)
C6—C7—C8—C3	59.44 (17)	C24—C26—C27—C28	−59.71 (17)
C10—C7—C8—C3	−60.78 (17)	C24—C26—C27—C29	60.39 (17)
C4—C5—C9—C1	−59.58 (17)	C26—C27—C28—C22	59.40 (17)
C6—C5—C9—C1	60.39 (17)	C29—C27—C28—C22	−60.76 (17)
C11—C1—C9—C5	−178.64 (13)	C23—C22—C28—C27	−59.40 (17)
C10—C1—C9—C5	−59.78 (17)	C21—C22—C28—C27	60.66 (17)
C2—C1—C9—C5	59.34 (16)	C26—C27—C29—C20	−60.15 (17)
C6—C7—C10—C1	−59.86 (18)	C28—C27—C29—C20	60.16 (17)
C8—C7—C10—C1	60.24 (17)	C30—C20—C29—C27	178.60 (13)
C11—C1—C10—C7	178.20 (13)	C25—C20—C29—C27	59.28 (17)
C9—C1—C10—C7	59.33 (17)	C21—C20—C29—C27	−58.88 (17)
C2—C1—C10—C7	−59.24 (17)	N7—N8—C30—O2	0.1 (2)
N2—N1—C11—O1	−7.1 (2)	N7—N8—C30—C20	−179.67 (12)
N2—N1—C11—C1	170.95 (13)	C25—C20—C30—O2	17.2 (2)
C10—C1—C11—O1	−5.6 (2)	C21—C20—C30—O2	138.20 (15)
C9—C1—C11—O1	113.00 (17)	C29—C20—C30—O2	−101.38 (17)
C2—C1—C11—O1	−126.86 (16)	C25—C20—C30—N8	−163.04 (13)
C10—C1—C11—N1	176.36 (14)	C21—C20—C30—N8	−42.05 (18)
C9—C1—C11—N1	−65.00 (17)	C29—C20—C30—N8	78.37 (16)
C2—C1—C11—N1	55.14 (18)	N8—N7—C31—C32	178.15 (13)
N1—N2—C12—C13	176.98 (14)	C34—N6—C32—C33	0.49 (17)
C15—N3—C13—C14	−0.20 (18)	C34—N6—C32—C31	−176.12 (14)
C15—N3—C13—C12	−179.78 (15)	N7—C31—C32—C33	−173.00 (17)
N2—C12—C13—C14	174.59 (17)	N7—C31—C32—N6	2.4 (2)
N2—C12—C13—N3	−6.0 (2)	N6—C32—C33—N5	−0.36 (18)
C15—N4—C14—C13	−0.44 (19)	C31—C32—C33—N5	175.66 (16)
C15—N4—C14—Cl1	178.68 (12)	N6—C32—C33—Cl2	−178.57 (12)
N3—C13—C14—N4	0.40 (18)	C31—C32—C33—Cl2	−2.6 (3)
C12—C13—C14—N4	179.91 (17)	C34—N5—C33—C32	0.08 (18)
N3—C13—C14—Cl1	−178.66 (12)	C34—N5—C33—Cl2	178.41 (12)
C12—C13—C14—Cl1	0.8 (3)	C33—N5—C34—N6	0.24 (17)
C14—N4—C15—N3	0.30 (19)	C33—N5—C34—C35	−179.45 (15)
C14—N4—C15—C16	−178.97 (16)	C32—N6—C34—N5	−0.47 (18)
C13—N3—C15—N4	−0.06 (19)	C32—N6—C34—C35	179.23 (15)
C13—N3—C15—C16	179.20 (15)	N5—C34—C35—C36	−63.0 (2)
N4—C15—C16—C17	84.5 (2)	N6—C34—C35—C36	117.35 (17)
N3—C15—C16—C17	−94.6 (2)	C34—C35—C36—C37	−56.7 (2)
C15—C16—C17—C18	76.1 (2)	C35—C36—C37—C38	−176.31 (15)

### Hydrogen-bond geometry (Å, °)

**Table:**

*D*—H···*A*	*D*—H	H···*A*	*D*···*A*	*D*—H···*A*
N1—H1···O3^i^	0.86 (1)	2.00 (1)	2.841 (2)	166 (2)
N3—H3···O1w^ii^	0.86 (1)	1.95 (1)	2.806 (2)	170 (2)
N6—H6···O2w	0.88 (1)	1.95 (1)	2.829 (2)	174 (2)
N8—H8···O3w^iii^	0.86 (1)	1.94 (1)	2.778 (2)	164 (2)
O3—H3o···O1w	0.84 (1)	1.84 (1)	2.673 (2)	177 (2)
O1w—H11···O1^ii^	0.84 (1)	2.00 (1)	2.821 (2)	166 (2)
O1w—H12···N5	0.84 (1)	1.91 (1)	2.751 (2)	175 (3)
O2w—H21···O2	0.85 (1)	2.07 (1)	2.905 (2)	172 (2)
O2w—H22···O2^iv^	0.84 (1)	1.93 (1)	2.764 (2)	176 (2)
O3w—H31···N4	0.84 (1)	1.94 (1)	2.773 (2)	169 (2)
O3w—H32···O2w	0.85 (1)	1.92 (1)	2.766 (2)	174 (2)

Symmetry codes: (i) −*x*+2, −*y*+2, −*z*+1; (ii) −*x*+1, −*y*+2, −*z*+1; (iii) *x*+1, *y*, *z*; (iv) −*x*+1, −*y*+2, −*z*.
